# Cardiovascular involvement in COVID-19: not to be missed

**DOI:** 10.21470/1678-9741-2020-0224

**Published:** 2020

**Authors:** Isadora S. Rocco, Walter J. Gomes, Marcela Viceconte, Douglas W. Bolzan, Rita Simone L Moreira, Ross Arena, Solange Guizilini

**Affiliations:** 1 Cardiology and Cardiovascular Surgery Disciplines, São Paulo Hospital, Escola Paulista de Medicina, Universidade Federal de São Paulo (Unifesp), São Paulo, SP, Brazil.; 2 Department of Human Motion Sciences, Physical Therapy School, Universidade Federal de São Paulo (Unifesp), Santos, SP, Brazil.; 3 Department of Physical Therapy, College of Applied Health Sciences, University of Illinois at Chicago, Chicago, IL, USA.

**Keywords:** COVID-19, Pneumonia, Viral, Extracorporeal Membrane Oxygenation, Myocarditis, Cardiovascular Diseases, Angiotensin Converting Enzyme 2, Mechanical Ventilation, SARS-CoV2

## Abstract

In December 2019, a striking appearance of new cases of viral pneumonia in Wuhan led to the detection of a novel coronavirus (SARS-CoV2). By analyzing patients with severe manifestations, it became apparent that 20 to 35% of patients who died had preexisting cardiovascular disease. This finding warrants the important need to discuss the influence of SARS-CoV2 infection on the cardiovascular system and hemodynamics in the context of clinical management, particularly during mechanical ventilation. The SARS-CoV2 enters human cells through the spike protein binding to angiotensin-converting enzyme 2 (ACE2), which is important to cardiovascular modulation and endothelial signaling. As ACE2 is highly expressed in lung tissue, patients have been progressing to acute respiratory injury at an alarming frequency during the Coronavirus Disease (COVID-19) pandemic. Moreover, COVID-19 leads to high D-dimer levels and prothrombin time, which indicates a substantial coagulation disorder. It seems that an overwhelming inflammatory and thrombogenic condition is responsible for a mismatching of ventilation and perfusion, with a somewhat near-normal static lung compliance, which describes two types of pulmonary conditions. As such, positive pressure during invasive mechanical ventilation (IMV) must be applied with caution. The authors of this review appeal to the necessity of paying closer attention to assess microhemodynamic repercussion, by monitoring central venous oxygen saturation during strategies of IMV. It is well known that a severe respiratory infection and a scattered inflammatory process can cause non-ischemic myocardial injury, including progression to myocarditis. Early strategies that guide clinical decisions can be lifesaving and prevent extended myocardial damage. Moreover, cardiopulmonary failure refractory to standard treatment may necessitate the use of extreme therapeutic strategies, such as extracorporeal membrane oxygenation.

**Table:** 

Abbreviations, acronyms & symbols				
ACE2 ARBs ARDS ARI CK COVID-19 CRP Cst ECMO etCO_2_/PaCO_2_ HEPA IMV LPV MERS-CoV NAb	= Angiotensin-converting enzyme 2 = Angiotensin receptor blockers = Acute respiratory distress syndrome = Acute respiratory infection = Creatine kinase = Coronavirus disease = C-reactive protein = Static compliance = Extracorporeal membrane oxygenation = End-tidal carbon dioxide/arterial carbon dioxide - dead space ratio = High-efficiency particulate air = Invasive mechanical ventilation = Lung-protective ventilatory strategy = Middle East Respiratory Syndrome = Neutralizing antibodies		NIV PaO_2_/FiO_2_ PBW PEEP R0 RAAS RV SARS-CoV2 ScVO_2_ SpO_2_/FiO_2_ V/Q VT WHO	= Noninvasive mechanical ventilation = Partial pressure arterial oxygen to inspired fraction of oxygen ratio = Predicted body weight = Positive end-expiratory pressure = Reproduction number = Renin-angiotensin-aldosterone system = Right ventricular = Severe scute respiratory syndrome coronavirus 2 = Central venous oxygen saturation = Pulse oximetric saturation to inspired fraction of oxygen ratio = Ventilation/Perfusion ration = Tidal volume = World Health Organization

## INTRODUCTION

In December 2019, a striking appearance of new cases of viral pneumonia in Wuhan preceded the detection of a novel coronavirus^[1]^. An alarming frequency of Severe Acute Respiratory Syndrome related to this novel coronavirus (SARS-CoV2)^[2]^ led to a rapid increase in the number of hospitalizations by the disease, referred to as Coronavirus Disease 2019 (COVID-19). A situation of Public Health Emergency of International Concern was alarmed by the World Health Organization (WHO) on January 30, 2020; the virus soon achieved worldwide spread and the declaration of a pandemic was made on March 11, 2020^[1]^. Although presenting with a low mortality rate, a unique characteristic of SARS-CoV2 is its transmission potential with a reproduction number (R0) of 2.24 to 3.58^[3]^, which is stronger than seasonal flu (R0=1.28) and previous pandemic viruses such as the 1918 Spanish flu (R0=1.80) and H1N1 in 2009 (R0=1.46)^[4]^. Moreover, a large percentage of infected individuals remain asymptomatic for a period of time and these events have led to a rapid and continuing increase in COVID-19 cases around the world. Viral mechanisms of human infection must be discussed to understand who the most exposed population is and guide public health decisions. 

The clinical presentation of SARS-CoV2 infection includes significant levels of systemic inflammation, with particular signs of leukopenia (25% of presentations), lymphopenia (63%), higher levels of D-dimer level, and elevated prothrombin time. Additionally, there have been reported markers of organ tissue damage, such as increased levels of aspartate aminotransferase (37%), and a rise in hypersensitive troponin I and creatine kinase (CK). Not only acute respiratory distress syndrome (ARDS) has been reported as a common complication (29%), but acute cardiac injury is also present in severe manifestations of SARS-CoV2 infection (12%)^[5,6]^. 

Several studies have demonstrated an association between an individual’s previous health status and severity of COVID-19 presentation^[5-7]^. The pre-existing medical risk factors appearing to be most ominous during the COVID-19 pandemic are hypertension, obesity, diabetes, previous chronic obstructive pulmonary disease, cancer, or cardiovascular disease^[8-10]^. By analyzing patients with severe manifestations and those who have died from SARS-CoV2 infection, 20 to 35% had a previous diagnosis of cardiovascular disease^[5,7,11]^. This trend underlines the need to discuss the influence of SARS-CoV2 infection on the cardiovascular system and hemodynamic considerations in patients requiring mechanical ventilation.

### How the SARS-CoV2 infection is related to cardiovascular features

The SARS-CoV2, similar to previous coronavirus strains, enters human cells through the spike protein binding to the angiotensin-converting enzyme 2 (ACE2)^[11]^. This enzyme participates in the angiotensin-converting process, which is an important component of cardiovascular modulation and endothelial signaling. Moreover, the ACE2 has an innate role in binding to cell membranes, which enables virus replication in the intracellular space^[11,12]^. As ACE2 is highly expressed in lung tissue, most of the manifestations of COVID-19 are related to respiratory symptoms. The ACE2 pathway of SARS-CoV2 largely affects the alveolar epithelial cells, leading to local damage and inflammation. These alterations evolve with increased capillary permeability, interstitial edema, and, therefore, thickening of the alveolar-capillary membrane. The infection mechanism of SARS-CoV2 explains the observation of acute lung injury and hypoxia in a significant number of cases. Also, the ACE2 tends to present higher levels in patients with cardiovascular disease, particularly because of the drugs used to control the renin-angiotensin-aldosterone system (RAAS) that usually increases the availability of this aminopeptidase^[12]^. 

The inflammatory response to SARS-CoV2 comprises two main stages^[13]^. The primary inflammatory response is mediated by apoptosis of epithelial and endothelial cells, releasing a vast proinflammatory content. Also, continuing viral replication causes a downregulation of ACE2 which reduces the protective role of this enzyme and leads to a dysfunction of the renin-angiotensin system with a further increase in vascular permeability and pulmonary cell infiltration. As macrophages and lymphocytes respond to the chemotaxis, there is a pyroptosis by internal replication of this virus, and this event explains the lymphopenia found in a significant number of patients. The secondary wave of the inflammatory response initiates with the expression of neutralizing antibodies (NAb) as an adaptive immunity; this stage is critical for patients to develop severe manifestations of COVID-19. The NAb activation enhances the Fc receptors related to IgG potentializing the inflammatory response. Why an increase in IgG expression exposes more the patients to a severe presentation of SARS-CoV2 infection is unclear. One possible hypothesis is that antibody-dependent enhancement of viral infection evolves into persistent viral replication and explains an overwhelming inflammatory response from macrophages^[12,13]^.

As described above, as the virus replication continues, it leads to a downregulation of ACE2, which is an important tissue protector^[14]^. Therefore, discussions about the pros and cons of withdrawing ACE inhibitors and angiotensin receptor blockers (ARBs) in cardiovascular patients have been made to reduce the risk of a severe COVID-19 course^[15,16]^. Further studies are necessary to understand whether these medications make patients more susceptible or whether its continuation has an essential protective role.

In this context, clinicians and scientists have distinguished cohorts of patients who are more susceptible to a systemic inflammatory condition during the COVID-19 pandemic. Although the pulmonary system is particularly at risk for injury due to the enhanced ACE2 expression, there is concern that other systems are also at risk for tissue injury, which will be discussed in subsequent sections.

### The pathway of pulmonary endangerment with SARS-CoV2

As described previously, the spike protein of SARS-CoV2 binds to ACE2 facilitating entry and replication, especially in pulmonary cells. It is important to highlight that ACE2 attached to the virus fails to adequately participate in the RASS and both events expose the lungs to a severe state of inflammation and vascular demodulation. These events explain the progression to ARDS observed during COVID-19^[16]^. Several reports about the clinical manifestation of COVID-19 demonstrate significant high levels of D-dimer level and prothrombin time^[5]^, which indicates a substantial coagulation disorder. Approximately 70% of non-survivors patients met the criteria of disseminated intravascular coagulation compared to only 0.6% of survivors. Furthermore, the timing of a marked increase of D-dimer and fibrin-related levels were associated with mortality risk^[14]^. Tang et al.^[17]^ administered anticoagulant therapy with low molecular weight heparin for 7 days or longer and yielded a mortality reduction of approximately 20% in patients with 6-fold D-dimer concentration and/or higher sepsis-induced coagulopathy score. In addition to SIRS, these assumptions suggest that systemic organic dysfunction could be also related to thromboembolic events at a microvascular level in pulmonary circulation. In this context, patients with COVID-19 are often hospitalized due to dyspnea, with some initially presenting with hypoxia and signs of respiratory failure. 

Oxygen supplementation is the main intervention for patients hospitalized with SARS-CoV2 infection; close monitoring for clinical deterioration is needed. The early institution of mechanical ventilation is a life-saving treatment that helps to support patients with pulmonary damage caused by COVID-19^[18]^. Noninvasive mechanical ventilation (NIV) was used in several cases in China and some other countries started shortly after hospital admission with a 30-minute efficacy evaluation^[18]^. Because this therapy evolves with a high risk of aerosolization and therefore is not recommended if the patient is not able to be isolated, if there is a shortage of adequate NIV interfaces, or if there is a non-double branch circuit with no environmental protection^[19]^. Moreover, while the use of a high flow nasal cannula has been proposed as a possible therapy to treat early hypoxia in these patients^[20]^, it must be used cautiously in patients with critically impaired levels of oxygenation due to the risk of therapy failure^[21]^. It is very important to understand that greater impairment levels of respiratory function require longer mechanical ventilation management that must be offered invasively. 

Clinical manifestation varies among the population, it is estimated that 30.8% of people infected with SARS-CoV2 had no symptoms^[22]^. Among symptomatic individuals, 15 to 20% require hospitalization, in which 5% progress to a critical condition needing more complex interventions. For those who ultimately present with symptoms, the median incubation time is approximately 5 days after infection^[19]^. Patients progressing to respiratory failure usually do so 4 to 6 days after initial symptoms appear (*i.e*., 9 to 10 days after initial infection), leading to an urgent need for invasive mechanical ventilation (IMV). The intubation procedure also comes with a high risk of aerosolization of the virus and thus steps must be taken to protect the staff performing this procedure from infection. Appropriate airborne/droplet individual protection equipment is required in this context, and no bag-valve-mask for ventilation is recommended. After intubation, the IMV has to be confirmed by adapting patients into ventilation through a double circuit protected with a high-efficiency particulate air (HEPA) filter^[16]^.

### Facing the challenges of invasive mechanical ventilation in COVID-19

Patients are reported presenting with significant reductions in oxygenation, with an partial pressure arterial oxygen to inspired fraction of oxygen ratio (PaO_2_/FiO_2_) frequently below 200. By the ARDS Berlin classification^[23]^, patients presenting with moderate to severe ARDS prompts the use of a lung-protective ventilatory strategy (LPV) with: 1) low tidal volume (VT) by predicted body weight (PBW); 2) low driving pressure (< 15cmH_2_O); and 3) preferential higher positive end-expiratory pressure (PEEP). Nevertheless, it has been reported that even in COVID-19 patients presenting with a low PaO_2_/FiO_2_, they still have a normal to near-normal static compliance (Cst)^[24]^. This has been intriguing respiratory therapists and clinicians around the world because we cannot conclude that COVID-19 leads to a typical ARDS. It seems that an overwhelming inflammatory state is responsible for a mismatching of ventilation and perfusion (V/Q), while a near-normal Cst may hide another important physiological explanation for low oxygenation^[5,24]^. 

#### Type 1: low PaO_2_/FiO_2_ and near-normal Cst

Patients with normal Cst underwent higher levels of PEEP may have more susceptibility to hemodynamic repercussions with possible tissue perfusion impairment^[25]^. Recent concern about using IMV strategies for typical ARDS extrapolated for COVID-19 patients led Gattinoni, Chiumello, and Rossi^[24]^ to describe different types of pulmonary injury in these patients. Around 50% of patients present with low PaO_2_/FiO_2_ but normal Cst, known as Type 1 patients. In these cases, lower VT in a range of 4 to 6 ml/kg is questionable and it would increase the necessity of higher breathing frequency to achieve target values of carbon dioxide arterial pressure (PaCO_2_). It would be reasonable to titrate PEEP levels avoiding alveolar distention by choosing better driving pressure values and controlling hemodynamic repercussions by monitoring sensitive measures of tissue perfusion. Combined with the fact that a restricted administration of volume has been recommended for COVID-19, patients can also be at a stage of lower intravascular volume. Accordingly, a possible hypovolemia state can enhance the susceptibility to pulmonary vessels collapse by higher levels of PEEP^[26]^. Therefore, the normality of intravascular volume is important to minimize the hemodynamic effects during positive pressure IMV. Independently of the disease condition, by applying IMV strategy, it is necessary to evaluate not only oxygenation but also hemodynamic status^[25,27]^. 

**Fig. 1:**
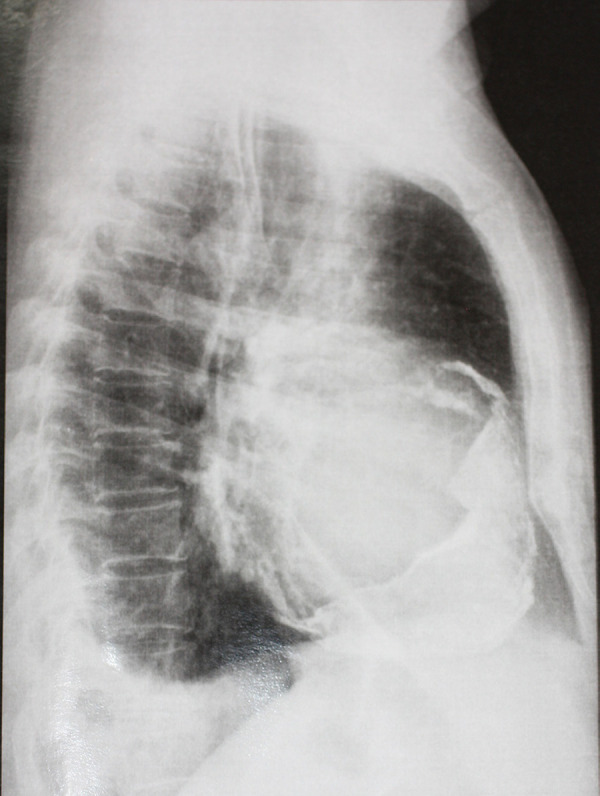
Flowchart of recommendation of MV management. Cst=static compliance; CVC=central venous catheter; DP=driving pressure; ECMO=extracorporeal membrane oxygenation; etCO2/PaCO2=Endtidal carbon dioxide/arterial carbon dioxide - dead space ratio; IMV=invasive mechanical ventilation; PaO2/FiO2=partial pressure arterial oxygen to inspired fraction of oxygen ratio; PBW=predicted body weight; PEEP=positive end-expiratory pressure; ScVO2=central venous oxygen saturation; SpO2/FiO2=pulse oximetric saturation to inspired fraction of oxygen ratio; SVC=superior venous cava; VT=tidal volume

Previous reports revealed substantial inaccuracy of hemodynamic status evaluation through macro signs such as mean arterial pressure and heart rate. Rivers et al.^[28]^ studied a cohort of sepsis patients and found that monitoring hemodynamics by central venous oxygen saturation (ScVO_2_) was much more precise to assess tissue perfusion and secure the success of sepsis treatment. Also, monitoring sepsis patients through ScVO_2_ leads to a 15% reduction in overall mortality. Indeed, lower values of ScVO_2_ are related to high expression of arterial lactate, and therefore, reflect circulatory inefficiency. Distorted values of ScVO_2_ predict a mismatch in oxygen offer and supply and may be occurring by low blood flow cardiac ejection, obstruction of the circulation, or hypovolemia. This measure is related to hidden hypoperfusion and low tissue oxygenation which evolves with further organic dysfunction. Several studies report that ScVO_2_ values lower than 60-70% or higher than 80% are related to higher mortality into several insights of critical care. However, it is important to highlight that arterial lactate is usually released when ScVO_2_ values fall below 50% ^[29]^. The authors of this review stress the necessity of paying closer attention to monitoring ScVO_2_ to assess hemodynamic repercussions during strategies of IMV (Figure 1). 

As described above, a pro-thrombotic characteristic presented in patients with SARS-CoV2 infection may silently affect pulmonary microcirculation. This event could help to explain the lung response of distorted V/Q and PaO_2_/FiO_2_ with a near-normal Cst. In pulmonary thromboembolism, the level of decrease in pulmonary Cst is related to the amount of edema and decrease in surfactant production^[30]^. A microvascular level of pulmonary infarction could not resonate with a very important decrease in Cst. Moreover, patients with type 1 manifestation of COVID-19 may evolve with V/Q mismatching due to impaired pulmonary blood flow distribution and regulation rather than collapse alveolar areas. 

In the context of COVID-19, there may be a significant frequency of micro thromboembolic events occurring in the lungs which could cause an increase in pulmonary vascular resistance. Combined with micro thromboembolic events, increasing PEEP could significantly augment right ventricular (RV) afterload, carrying hemodynamics repercussions and diminishing tissue perfusion. A superior strategy to investigate the imbalance between oxygen offering and demand would be by evaluating ScVO_2_, guaranteeing RV protection, and tissue perfusion^[25,27,29]^. These observations are important to discuss the responsiveness of prone positioning in patients with COVID-19 type 1 lung injury. A PaO_2_/FiO_2_ ratio below 150 is a recommendation to adopt a prone position in COVID-19^[16]^. Nevertheless, a near-normal Cst with a low PaO_2_/FiO_2_ may indicate that a further improvement in oxygenation may be related to blood flow redistribution rather than the classic opening of collapsed alveolar units. Since type 1 COVID-19 patients respond to prone positioning for circulatory reasons, at least a small decrease in oxygenation after return to supine is expected. Still, this strategy has been recommended as a rescue therapy in type 1 COVID-19 to guarantee more time to fight against SARS-CoV2 infection^[24]^.

#### Type 2: low PaO_2_/FiO_2_ and low Cst

Approximately 20 to 30% of patients with SARS-CoV2 infection may present with a type 2 manifestation of pulmonary impairment. As Gattinoni et al.^[24]^ suggest, type 2 patients present with hypoxia associated with Cst <40 ml/cmH_2_O, as a typical ARDS. The specific pathway that explains the clinical course to different phenotypes is not yet described, but initial thoughts are it may entail increased edema or be related to patient self-inflicted ventilator-induced lung injury due to initial respiratory management. Before IMV, several patients with hypoxia present with vigorous levels of inspiratory effort and a greater amount of negative intrathoracic pressures, at which some of them received NIV. These type 2 patients manifest more expressive non-aerated lung tissue by radiologic imaging, lower Cst, and high right-to-left shunt. Pinsky et al.^[25]^ identified that adding positive pressure IMV will increase ScVO_2_ and consequent PaO_2_ if right-to-left shunt exists. Type 2 patients would benefit from an LPV strategy with lower levels of VT and a higher level of PEEP, a typical approach for ARDS. Besides, as a thrombotic microvascular event and hypovolemia may be occurring, ScVO_2_ monitoring during IMV strategies is important to avoid silent tissue hypoperfusion and protect from increased RV afterload, especially during more aggressive treatment strategies. As in typical ARDS, type 2 patients with PaO_2_/FiO_2_ below 150 probably will benefit more from prone positioning in a period of at least 16 hours. The authors of this review highlight again the importance of evaluating ScVO_2_ before and during the adoption of this strategy to avoid tissue damage and cardiac overload.

### Tracheal cuff management as part of a lung-protective strategy

Complications associated with mechanical ventilation have been widely described in the literature. For this reason, strategies as lung-protective ventilation have been adopted to avoid ventilator-induced lung injury^[31]^. Recently, authors have been reported some indirect adverse events secondary to mechanical ventilation, including tracheal injury^[32]^. For them, lung-protective mechanical ventilation would not be limited to mechanical ventilator adjustment, but also associated with intubation/extubation complications^[32]^. In these circumstances, cuff pressure management plays an important role to prevent complications: excessive cuff inflation could cause ischemic damage to tracheal mucosa; insufficient cuff inflation could promote subglottic secretion aspiration leading to pneumonia, also air escape around the cuff could negatively impact in prescribed tidal volume^[33]^. 

In Covid-19 patients, cuff management assumes a large importance, once the air escape secondary to cuff underinflation could generate aerosols spread, predisposing contamination, and compromising the health staff safety. Safe limits and therapeutic range for cuff pressure adjustment is well described in literature^[33]^. Also the literature recommends cuff inflation until air leak stops, but this exact moment is hard to be found. The volume-time curve could be an alternative for endotracheal tube cuff management in this scenario of Covid-19. Volume-time curve allows to find the exact volume to fill the cuff and seal the airway, ensuring the prescribed tidal volume and avoiding the aerosol spread^[34]^. Monitoring cuff inflation by volume-time curve is safe and simple: the descendant branch of the curve needs to reach the baseline, if the expired volume is smaller than the inspired one, it means that an air leakage is occurring; air leak is represented by a flattening of the curve. If an air leak is identified we only need to inflate the cuff until the descendant branch of the curve reaches the baseline^[33]^. 

### How COVID-19 is related to myocardial injury?

Recent reports have observed an elevation of brain natriuretic peptide, CK, and in some cases, increased troponin I in patients with SARS-CoV2 infection^[35,36]^. These observations reveal a myocardial injury during severe manifestations of the disease. It is well established that a severe respiratory infection and a scattered inflammatory process can cause non-ischemic myocardial injury, including the evolution to myocarditis. The presence of myocardial injury was also detected as electrocardiographic and echocardiographic abnormalities. This event is expressed in up to 17% of total hospitalized patients during COVID-19, and up to 59% in cases of death^[2,37]^.

Recent reports have identified the occurrence of myocarditis with a global decrease in ventricular function, observed by lower left ventricular ejection fraction in patients with SARS-CoV2 infection. In a cohort of 150 patients, Ruan et al.^[36]^ identified that myocardial damage/heart failure contributes to death combined with respiratory failure in 33% of cases and myocardial damage with circulatory failure in 7%. Moreover, Liu et al.^[38]^ reported a case of fulminant myocarditis and, interestingly, pathological investigations of this case report did not find an obvious histological finding, such as nuclear or cytoplasmic viral inclusions into myocardial fibers. This observation suggests that heart damage may not be directly related to the SARS-CoV2 replication in the myocardial tissue^[39]^. It may be possible that myocardial injury is the result of a supply-demand mismatch and additional damage caused by microembolization of the coronary arteries, triggering peripheral and silent myocardial ischemia. Nonetheless, these assumptions must be investigated in further histological investigations and future cohort monitoring. 

Finally, acute respiratory infection (ARI) severity may be related to an increased risk of a classic myocardial infarction event. It is well known that greater inflammatory responses in the endothelium could enhance susceptibility to the rupture of unstable atherosclerotic plaques. Therefore, the incidence of myocardial infarction among cardiovascular disease patients is increased after a viral ARI, including the coronavirus (Incidence Ratio: 3.30 [1.90-5.73)]^[40]^. We also suggest a closer monitoring of cardiocirculatory function in severe cases of ARI, especially in patients with precedent cardiovascular disease. Therefore, the lethality of COVID-19 is related to cardiac failure with subsequent multiple organ dysfunction. Early strategies that guide clinical decisions can be lifesaving and prevent myocardial damage.

### How to manage refractory gas-exchange failure associated with myocardial injury?

Over the years, patients with a refractory gas-exchange impairment in the setting of severe ARDS may be rescued with an extreme therapeutic strategy, the extracorporeal membrane oxygenation (ECMO). ECMO therapy in adults was first applied in the 1970s and has become an alternative life-support technique for the management of life-threatening pulmonary and/or cardiac failure^[41]^. ECMO provides blood flow rates to support gas exchange (venovenous access) or circulatory support (venoarterial access). ECMO support is provided through a vascular access cannula inserted in a central vein, connected to a blood pump that diverts blood, under negative pressure, to a gas exchange device, known as an oxygenation membrane. After gas exchange occurs in the oxygenation membrane, blood can return to the right atrium or close to it through a second vascular cannula, in the case of venovenous support^[42]^.

In patients with severe ARDS, there is a general consensus on an initial approach that includes IMV with low tidal volumes and the use of optimal values of PEEP to avoid alveoli collapse^[23]^. However, IMV may potentiate lung injury due to overdistention and repetitive opening and closing off the lung units. ECMO has been indicated in patients with severe respiratory failure to temporarily provide gas exchange, leading to less aggressive IMV, reducing pulmonary damage^[43]^. 

The conventional ventilation or ECMO for ARDS (CESAR) trial was the first contemporary randomized trial to investigate the safety and efficacy of ECMO in patients with acute respiratory failure in adults compared to conventional mechanical ventilation care^[41,44]^. Patients randomized to the ECMO group were transferred to an ECMO center. The authors concluded that ECMO was a clinically effective treatment for ARDS, with low mortality in the ECMO group compared to conventional treatment, as well as the outcomes after a six-month follow-up. In the same way, Beurtheret et al.^[45]^, in a retrospective analysis, demonstrated good results with venovenous ECMO for patients with H1N1-associated respiratory failure, with an in-hospital mortality rate of 17%. Similarly, Cianchi et al.^[46]^, in a case series, suggested that ECMO is a safe and feasible therapy for patients with H1N1-induced ARDS.

More recently, in 2012, a novel coronavirus was detected and associated with Middle East Respiratory Syndrome (MERS-CoV). MERS-CoV was related to significant mortality due to respiratory failure with refractory hypoxemia, multiorgan failure, and septic shock. Due to the severity of the manifestations, ECMO also proved to be a therapeutic alternative, as noted by Alshahrani et al.^[47]^ in a retrospective cohort study. The authors found that ECMO was associated with lower in-hospital mortality, better oxygenation, and fewer organ failure episodes compared to conventional therapy in patients with severe MERS-CoV. 

In the COVID-19 population, recent data provide some insights into ECMO utilization^[35,48]^. Among 6 patients who underwent ECMO, five (83%) died. Since the inflammatory aggression of SARS-CoV2 infection leads to a potentially disseminated coagulation disturbance and an expressive decrease of lymphocytes and platelets, the use of ECMO should be considered. During ECMO, an inherent immunological activation occurs secondary to blood cell exposure to a non-endothelialized circuit. Moreover, it is necessary to establish rigorous control of coagulation to avoid clots through the membrane. For these reasons, the cost-benefit of ECMO therapy is a tough clinical decision that demands frequent laboratory monitoring and staff with expertise. Risnes et al.^[49]^ identified that higher levels of interleukin-6 (IL-6) predicts mortality in patients receiving ECMO. Elevated IL-6 and C-reactive protein (CRP) were observed in deceased patients with COVID-19, suggesting that SARS-CoV2 causes a “cytokine storm syndrome”. Therefore, we suggest monitoring of inflammatory markers such as IL-6 when available and close monitoring of CRP and white blood cells counts. 

ECMO is usually related to higher rates of mortality, especially because it is often used as a life-saving rescue therapy in patients who already have the severest manifestation of a pulmonary condition^[48]^. A great point of discussion should be the timing of this interventional approach and availability of a specialized staff^[50]^. Once prone positioning fails to lead to a great amount of improvement, further studies are necessary to evaluate the timing and efficacy of ECMO administration in refractory cases.

## CONCLUSION

The COVID-19 pandemic is proving to be one of the most challenging health issues of our time. In severe cases requiring hospitalization and advanced interventional measures (*e.g*, MV), tissue perfusion monitoring by ScVO_2_ during high levels of PEEP and Intensive Care Unit treatment are recommended. More complex strategies should be considered in extreme cases requiring the commitment of ECMO referral centers/staff and overall better management of the growing number of cases during COVID-19.

**Table:** 

Authors' roles & responsibilities	
ISR WJG MV DWB RSLM RA SG	Substantial contributions to the conception or design of the work; or the acquisition, analysis, or interpretation of data for the work; drafting the work or revising it critically for important intellectual content; final approval of the version to be published Substantial contributions to the conception or design of the work; or the acquisition, analysis, or interpretation of data for the work; drafting the work or revising it critically for important intellectual content; final approval of the version to be published Acquisition, analysis, or interpretation of data for the work; final approval of the version to be published Acquisition, analysis, or interpretation of data for the work; final approval of the version to be published Acquisition, analysis, or interpretation of data for the work; final approval of the version to be published Drafting the work or revising it critically for important intellectual content; final approval of the version to be published Substantial contributions to the conception or design of the work; or the acquisition, analysis, or interpretation of data for the work; drafting the work or revising it critically for important intellectual content; final approval of the version to be published
